# Associations between theory of mind and clinical symptoms in recent onset schizophrenia spectrum disorders

**DOI:** 10.3389/fpsyt.2023.1044682

**Published:** 2023-02-09

**Authors:** Audrey Cayouette, Élisabeth Thibaudeau, Caroline Cellard, Marc-André Roy, Amélie M. Achim

**Affiliations:** ^1^École de Psychologie, Université Laval, Québec, QC, Canada; ^2^Centre de recherche CERVO, Québec, QC, Canada; ^3^Department of Psychiatry, McGill University, Montreal, QC, Canada; ^4^Département de psychiatrie et de neurosciences, Université Laval, Québec, QC, Canada

**Keywords:** social cognition, theory of mind, clinical symptoms, schizophrenia spectrum disorders, PANSS five-factor model

## Abstract

**Introduction:**

People with schizophrenia often present with Theory of mind (ToM) deficits, and the link between these deficits and clinical symptoms remains to be refined, for instance through the use of more recent assessment methods. The objective of this study was to examine the associations between a psychometrically sound ToM task and the clinical symptoms of schizophrenia as measured with the five dimensions of the Positive and Negative Syndrome Scale (PANSS) namely positive, negative, cognitive/disorganization, depression/anxiety and excitability/hostility, while controlling for non-social cognitive abilities.

**Methods:**

Seventy participants with recent-onset schizophrenia spectrum disorders (SSD) were assessed for ToM using the Combined stories task (COST) and for clinical symptoms using the PANSS.

**Results:**

The results revealed significant correlations between ToM and the positive (*r* = −0.292, *p* = 0.015) and cognitive/disorganization (*r* = −0.480, *p* < 0.001) dimensions when controlling for non-social cognitive abilities. In contrast, the negative symptoms dimension was only significantly correlated with ToM when non-social cognitive abilities were not controlled for (*r* = −0.278, *p* = 0.020).

**Discussion:**

Very few prior studies used the five-dimensions of the PANSS to examine the link with ToM and this study is the first to rely on the COST, which includes a non-social control condition. This study highlights the importance of taking non-social cognitive abilities into account when considering the relationship between ToM and symptoms.

## Introduction

Schizophrenia spectrum disorders (SSD) are associated with a reduction in social connections and rate of employment as well as an impaired ability to live independently (1). There is thus a major interest in further understanding the range of factors that contribute to these difficulties as well as their inter-relationships. Clinical symptoms, and more specifically negative symptoms can affect functional outcomes and quality of life (2). Clinical symptoms are traditionally divided into three dimensions namely positive symptoms (e.g., hallucinations, delusions), disorganization (e.g., disorganized speech or behavior, inappropriate affect) and negative symptoms (e.g., blunted affect, a sociality) (3).

In addition to clinical symptoms, cognitive deficits have gained increased interest in SSD in the last decades given their important association with functioning difficulties (4, 5), with a particular interest for social cognition in the last few decades. Social cognition refers to the mental processes underlying social interactions including perceiving, interpreting and generating responses to the intentions, dispositions and behaviors of others (6, 7). Theory of mind (ToM), one of the core domains of social cognition, is defined as the ability to represent and infer the mental states of others, such as their intentions, emotions, or beliefs (7, 8). ToM deficits in people with schizophrenia have been observed during all phases of the illness (9, 10), and show a significant association with community functioning and social skills (4, 5) as well as other neurocognitive functions (11).

Overall, clinical symptoms and ToM are key variables to consider to promote functional recovery in people with SSD, and numerous studies have reported an association between these two groups of constructs (12–14). The interest for the associations between ToM and symptoms was initially driven by the cognitive theory from Frith (15) suggesting that ToM deficits could lead to disorders of willed action, other-monitoring and self-monitoring. According to this theory, disorders of willed action (apathy and bizarre behavior) emerge from the individual's inability to perceive their own intentions. Disorders of other-monitoring could explain a reduced awareness of other's mental state, leading to disorganized communication for example, while a diminished awareness of self-generated thoughts could lead to auditory hallucinations and delusions (15, 16). Supporting that claim, Mehl et al. (13) showed that patients with persecutory delusions were specifically impaired on a ToM task when compared to patients with remitted delusions and non-clinical control participants. Several other studies have also targeted the links between ToM and clinical symptoms, but overall this literature is heterogenous in terms of its methods and results, with some studies finding a significant link between ToM and either positive, negative and/or cognitive/disorganization symptoms (13, 17, 18). A recent meta-analysis quantified and compared the strength of the associations between ToM and the clinical symptoms in a population of SSD (19). They found small to moderate associations between ToM and cognitive/disorganization symptoms (Zr = 0.28), and between ToM and negative symptoms (Zr = 0.24), these associations being significantly stronger than with the other symptom dimensions. When looking at the individual symptoms, Difficulty in abstract thinking (Zr = 0.36) and Conceptual disorganization (Zr = 0.39) showed the strongest associations with ToM. Only a small association (Zr = 0.16) was found between ToM and positive symptoms (19). Interestingly, the meta-analysis by Thibaudeau et al. (19) observed stronger associations for the studies that relied on ToM tasks targeting a combination of mental states. Moreover, the associations between ToM and clinical symptoms were stronger in studies with younger patients.

A few studies also explored the associations between ToM and clinical symptoms in a population with recent onset SSD, again with heterogenous findings including significant correlations between ToM and the negative (14, 20) and the cognitive/disorganization symptom (10, 14) dimensions. The association with the positive symptoms dimension was also less consistent in this population (20–23).

While these results in first episode and more chronic participant samples support possible associations between ToM and symptoms of SSD, their inconsistencies regarding the symptom dimensions associated with ToM prevent a clear understanding of the specific relationships between these two domains. Prior to the meta-analysis of Thibaudeau et al. (19), a narrative review on the subject considered 30 studies that explored the associations between ToM and clinical symptoms (16), and the author concluded that it was not possible to identify one particular symptom or cluster of symptoms associated with ToM. They suggested that this lack of clear results stemmed from the variability in the methods (e.g., ToM tasks or symptom classification) used between the different studies and therefore, the variability in the results (10, 16).

The studies that thus far examined the relationship between ToM and clinical symptoms suffered from two important limitations that may partly explain the heterogeneity of their results. First, in most prior studies targeting this relationship, symptoms were assessed and regrouped using the original three subscales of the *Positive and Negative Syndrome Scale* (PANSS), i.e., positive, negative and general psychopathology, which factor analyses have repeatedly rejected, rather yielding a five factor solution (24, 25). For instance, the original three-subscale version of the PANSS did not include the cognitive/disorganization, anxiety/depression and excitability/hostility dimensions identified in factor analyses. Moreover, the positive and negative dimensions in the five-factor model do not totally overlap with those included in the original three-subscale version. Hence, the relationships between ToM and either of these rigorously defined symptom dimensions remain to be further studied.

Second, these previous studies have typically assessed ToM with tasks plagued with questionable psychometric properties (26, 27). For instance, the ToM tasks used included an insufficient number of items and/or presented with important ceiling effects that may hamper the detection of subtle ToM deficits (27, 28) and/or did not include control conditions needed to disentangle specific ToM deficits vs. deficits stemming from general neurocognitive impairments (29). Those psychometric limitations may then compromise the validity and the reproducibility of the results (27, 30). Given these two limitations, it is impossible to conclude about the specificity and the strength of the associations between symptom dimensions and ToM performance.

The main objective of this study was to examine the associations between ToM and the clinical dimensions of SSD using the five dimensions of the PANSS (positive, negative, cognitive/disorganization, depression/anxiety and excitability/hostility symptoms) and a psychometrically sound ToM task, the *Combined stories task* (8, 31), in a population with recent onset SSD, while controlling for non-social cognitive abilities. An additional exploratory objective was to examine the associations between ToM and the individual symptoms included in the five dimensions of the PANSS. A better understanding of these relationships in SSD could help identify more precise targets for intervention in this population. Based on the literature that was available before we performed our analyses, we initially expected a significant association between ToM and negative symptoms as well as positive symptoms (12–14). In addition, a significant association was also expected between ToM and cognitive/disorganization symptoms due to the well-documented associations between ToM and non-social cognition (11, 32).

## Method

### Participants

Seventy (33) participants with recent onset SSD (mean age = 26.1 years, SD = 4.6; 61 men) were included in this study. Data were obtained from an existing database created in the context of a larger study (8, 29, 34, 35) including young adults with a recent-onset SSD.

Participants were recruited from the Clinique Notre-Dame des Victoires (Quebec City, Canada), a specialized outpatient clinic that provides intensive and multidimensional care to young adults (18 to 35 years old) in the early stages of a psychotic disorder. The mean duration of illness for the current sample was 19.2 months (range from 1 to 72 months). The duration of illness was calculated as the time between the first antipsychotic medication and the testing. All patients were considered clinically stable by the treating psychiatrist at the time of recruitment. Additional demographic and clinical characteristics are displayed in Table 1, which shows that patients exhibited mild symptoms (mean total PANSS score of 54.5) (36). Diagnoses were established by the treating psychiatrist through a best-estimate diagnosis procedure, based on a comprehensive semi-structured interview (see section *Clinical assessment*), family history as well as direct reports from the rest of the clinical team and medical reports (37). For the ToM abilities, when compared to norms based on a sample of 84 healthy controls (29), the average performance of the patients in the current study was below average for the ToM items (QM; Z score = −1.50) and in low average for the non-social reasoning items (QR; Z score = −0.69). Patients with an estimated intellectual quotient under 70, as assessed with a dyad form of the Wechsler Adult Intelligence Scale, Vocabulary and Block design subtests (38), and those with a history of neurological disorder were excluded. The study was approved by the local ethics committee (CER CIUSSS-CN) and all participants gave informed consent after the study procedures were explained.

**Table 1:** Clinical and demographic characteristics of the sample.

***N* = 70**	***N***	**Mean**	**Standard deviation**	**Range**
Men	61			
Women	9			
Age		26.1	4.6	18–37
Duration of illness (months)		19.2	19.2	0–2
**PANSS scores per dimension**				
Positive dimension (/42)		14.9	5.1	6–30
Negative dimension (/49)		16.3	6.4	7–30
Cognitive/disorganization dimension (/35)		9.4	3.1	5–18
Depression/anxiety dimension (/28)		7.9	2.6	4–15
Excitability/hostility dimension (/28)		6.0	2.5	4–13
PANSS total score (/182)		54.7	13,3	30–93
**COST**				
ToM items (/52)		38.1	7.3	15–48
Non-social reasoning items (/12)		10.0	1.8	4–12
**Diagnosis**				
Schizophrenia	51			
Schizoaffective disorder	10			
Delusional disorder	6			
Psychosis not otherwise specified	2			
Schizophreniform disorder	1			
**Principal antipsychotic**				
Quetiapine	26			
Risperidone	16			
Aripiprazole	13			
Clozapine	7			
Olanzapine	6			
Paliperidone	1			
None	1		

PANSS, Positive and Negative Syndrome Scale; ToM, theory of mind; COST, Combined stories test.

## Material

### Clinical assessment

A comprehensive semi-structured interview was used for the clinical assessment that was based on the Structured Clinical Interview for DSM-IV (SCID-IV) (39) as well as the Structured Clinical Interview for the PANSS (SCI-PANSS) (40) and included questions from several additional instruments [see the Supplement from Achim et al. (29) for a full list]. The *Positive and Negative Symptoms Scale* (3) was then used to rate each symptoms on a Likert scale ranging from one (absent) to seven (extreme) and the symptoms were classified into the five following dimensions: positive (e.g., delusions, hallucinatory behavior, suspiciousness), negative (e.g., blunted affect, poor rapport, emotional withdrawal), cognitive/disorganization (e.g., abstraction, mannerism, disorientation), depression/anxiety (e.g., somatic concern, anxiety, guilt feelings) and excitability/hostility (e.g., hostility, poor impulse control, uncooperativeness) according to the classification of Lehoux et al. (24) (the full list of symptoms within each dimension can be found in Table 2).

**Table 2:** Association between ToM, ToM controlling for non-social reasoning, Non-social reasoning, and the clinical symptoms of schizophrenia.

	**ToM controlling for** **non-social reasoning**	**ToM**	**Non-social reasoning**
**Positive symptoms dimension**	***r*** **=** **−0.29**, ***p*** **=** **0.015**^**a**^	*r* = −0.24, *p* = 0.047	*r* = 0.06, *p* = 0.612
P1. Delusions	*r* = −0.23, *p* = 0.053	*r* = −0.19, *p* = 0.122	*r* = 0.06, *p* = 0.629
P3. Hallucinatory behavior	*r* = −0.19, *p* = 0.127	*r* = −0.17, *p* = 0.166	*r* = 0.00, *p* = 0.995
P5. Grandiosity	*r* = −0.10, *p* = 0.404	*r* = −0.08, *p* = 0.521	*r* = 0.03, *p* = 0.781
P6. Suspiciousness and persecution	*r* = −0.07, *p* = 0.558	*r* = −0.05, *p* = 0.693	*r* = 0.04, *p* = 0.744
G9. Unusual thought content	*r* = −0.21, *p* = 0.084	*r* = −0.14, *p* = 0.249	*r* = 0.12, *p* = 0.342
G12. Lack of judgment and insight	***r*** **=** –**0.34**, ***p*** **=** **0.004**^**a**^	*r* = −0.31, *p* = 0.009	*r* = 0.06, *p* = 0.604
**Negative symptoms dimension**	*r* = −0.20, *p* = 0.092	***r*** **=** –**0.28**, ***p*** **=** **0.020**^**a**^	*r* = −0.23, *p* = 0.054
N1. Blunted affect	*r* = −0.04, *p* = 0.728	*r* = −0.06, *p* = 0.605	*r* = −0.06, *p* = 0.636
N2. Emotional withdrawal	*r* = −0.19, *p* = 0.116	***r*** **=** –**0.28**, ***p*** **=** **0.018**^**a**^	*r* = −0.27, *p* = 0.024
N3. Poor rapport	*r* = −0.27, *p* = 0.024	***r*** **=** –**0.34**, ***p*** **=** **0.005**^**a**^	*r* = −0.23, *p* = 0.059
N4. Passive/apathetic social withdrawal	*r* = −0.29, *p* = 0.016	***r*** **=** –**0.36**, ***p*** **=** **0.002**^**a**^	*r* = −0.26, *p* = 0.031
N6. Lack of spontaneity	*r* = −0.20, *p* = 0.106	*r* = −0.24, *p* = 0.050	*r* = −0.14, *p* = 0.247
G7. Motor retardation	*r* = −0.15, *p* = 0.228	*r* = −0.23, *p* = 0.052	*r* = −0.24, *p* = 0.042
G16. Active social avoidance	*r* = 0.04, *p* = 0.760	*r* = −0.00, *p* = 1.000	*r* = −0.08, *p* = 0.513
**Cognitive/Disorganization dimension**	***r*** **=** –**0.40**, ***p*** **<** **0.001**^**a**^	***r*** ** = −0.48**, ***p*** **<** **0.001**^**a**^	***r*** **=** –**0.32**, ***p*** **=** **0.006**^**a**^
P2. Conceptual disorganization	***r*** **=** –**0.30**, ***p*** **=** **0.013**^**a**^	***r*** **=** –**0.32**, ***p*** **=** **0.007**^**a**^	*r* = −0.13, *p* = 0.288
N5. Difficulty in abstract thinking	***r*** **=** –**0.44**, ***p*** **<** **0.001**^**a**^	***r*** **=** –**0.55**, ***p*** **<** **0.001**^**a**^	***r*** **=** –**0.45**, ***p*** **<** **0.001**^**a**^
G5. Mannerism and posturing	*r* = −0.14, *p* = 0.266	*r* = −0.10, *p* = 0.403	*r* = 0.05, *p* = 0.683
G10. Disorientation	*r* = −0.03, *p* = 0.782	*r* = −0.05, *p* = 0.671	*r* = −0.05, *p* = 0.686
G11. Poor attention	*r* = −0.23, *p* = 0.055	***r*** **=** –**0.33**, ***p*** **=** **0.005**^**a**^	***r*** **=** –**0.31**, ***p*** **=** **0.009**^**a**^
**Depression/Anxiety dimension**	*r* = 0.06, *p* = 0.633	*r* = 0.08, *p* = 0.488	*r* = 0.07, *p* = 0.542
G1. Somatic concern	*r* = −0.07, *p* = 0.545	*r* = −0.04, *p* = 0.743	*r* = 0.06, *p* = 0.601
G2. Anxiety	*r* = 0.11, *p* = 0.377	*r* = 0.18, *p* = 0.144	*r* = 0.19, *p* = 0.116
G3. Guilt feelings	*r* = 0.08, *p* = 0.523	*r* = 0.08, *p* = 0.519	*r* = 0.02, *p* = 0.883
G6. Depression	*r* = 0.05, *p* = 0.670	*r* = 0.01, *p* = 0.911	*r* = −0.08, *p* = 0.518
**Excitability/hostility dimension**	*r* = −0.05, *p* = 0.691	*r* = 0.01, *p* = 0.921	*r* = 0.13, *p* = 0.279
P4. Excitement	*r* = 0.01, *p* = 0.971	*r* = 0.04, *p* = 0.773	*r* = 0.07, *p* = 0.548
P7. Hostility	*r* = 0.04, *p* = 0.744	*r* = 0.08, *p* = 0.507	*r* = 0.11, *p* = 0.387
G8. Uncooperativeness	*r* = −0.16, *p* = 0.192	*r* = −0.10, *p* = 0.428	*r* = 0.11, *p* = 0.365
G14. Poor impulse control	*r* = 0.03, *p* = 0.833	*r* = 0.09, *p* = 0.460	*r* = 0.16, *p* = 0.195

^a^Significant even after applying the FDR correction for multiple comparisons. Values in bold mean the same thing as values followed by the letter a, meaning that they are significant.

### ToM assessment

ToM was assessed with the *Combined Stories Test* (8, 31). In this verbal ToM task, participants read 30 stories aloud and for each story, they are asked to answer one or two questions targeting the mental states (e.g., emotions, beliefs or intentions) of the story characters (ToM items). Six non-social reasoning items are also included and require non-social reasoning (i.e., general reasoning abilities) rather than mental states attributions. The answers are rated 0, 1, or 2 points according to a validated scoring grid that takes into account the accuracy and completeness of the answers (e.g., one point is given for a concrete, incomplete answer; zero point for an incorrect answer) for a total of 52 points for the ToM items and 12 points for the non-social reasoning items. The participant can refer to the stories at any time during the test. Each story (ToM and non-social reasoning items) also includes control questions, scored zero or one point, to control for any attention or reading problem during the task. The COST presents with good psychometric properties, including validity (8) and reliability (8, 31). More specifically, Thibaudeau et al. (31) evaluated the psychometric properties of the COST in a group of healthy adults and reported excellent inter-rater reliability and good test-retest reliability (31). Another study in a sample of thirty-one participants with first episode psychosis (8) reported adequate convergent validity with Sarfati's cartoon task (41), a good internal consistency and an excellent inter-rater reliability. In addition, both prior studies (8, 31) reported normal distributions and no ceiling effect for the COST.

### Statistical analyses: Main objective

Normality of the distributions was first confirmed for the ToM items of the COST and the positive, negative, cognitive/disorganization, and the depression/anxiety dimensions of the PANSS. For the other, non-normally distributed variables, logarithmic transformations were applied before further analyses.

Partial correlations were performed to examine the associations between the five dimensions of the clinical symptoms and ToM performance while controlling for general reasoning abilities (non-social reasoning items from the COST). To ease the comparison between our results and those from previous studies, Pearson correlations (without the covariate) were also performed between the five dimensions of the clinical symptoms and ToM. For completeness, Pearson correlations were also performed to examine the associations between the five dimensions of the clinical symptoms and non-social reasoning. For each of these each analyses, a False Discovery Rate (FDR) correction was applied to control for multiple correlations (42).

### Statistical analyses: Exploratory objective

Partial correlations were performed to examine the associations between the individual symptoms within each PANSS dimension and ToM performance while controlling for non-social reasoning. Again, to allow comparison with the results of previous studies, Pearson correlation were performed between individual symptoms and ToM performance. Finally, Pearson correlations were also performed to examine the associations between the individual symptoms and non-social reasoning. A False Discovery Rate (FDR) correction was applied to control for multiple correlations within each of the five PANSS dimensions (42).

## Results

The full set of results is presented in Table 2 and the significant associations between symptoms and ToM are detailed below.

### Main objective

For the positive symptoms dimension, the association with ToM was significant only when controlling for non-social reasoning (*r* = −0.29, *p* = 0.015) and fell below the statistical threshold without the covariate (*r* = −0.24, *p* = 0.047). For the negative symptoms dimension, the opposite pattern was observed, i.e., the correlation was significant without the covariate (*r* = −0.28, *p* = 0.020), and fell below the statistical threshold with the covariate (*r* = −0.20, *p* = 0.092). Finally, for the cognitive/disorganization symptoms dimension, a significant negative correlation with ToM was observed both when non-social reasoning was controlled for (*r* = −0.40, *p* < 0.001) and without the covariate (*r* = −0.48, *p* < 0.001).

### Exploratory objective

Within the positive symptoms dimension, Lack of judgement and insight showed a significant negative correlation with ToM when controlling for non-social reasoning (*r* = −0.34, *p* = 0.004), but fell below the FDR threshold without the covariate (*r* = −0.31, *p* = 0.009). Within the negative symptoms dimension, no association reached the significance threshold when controlling for non-social reasoning. However, Emotional withdrawal (*r* = −0.28, *p* = 0.018), Poor rapport (*r* = −0.34, *p* = 0.005) and Passive/apathetic social withdrawal (*r* = −0.36, *p* = 0.002) showed a significant correlation with ToM without the covariate. For the cognitive/disorganization symptoms dimension, Conceptual disorganization (*r* = −0.30, *p* = 0.013) and Difficulty in abstract thinking (*r* = −0.44, *p* < 0.001) were significantly correlated with ToM when controlling for non-social reasoning, and these correlations remained significant after removing the covariate [respectively (*r* = −0.32, *p* = 0.007) and (*r* = −0.55, *p* < 0.001)]. Poor attention only reached significance without the covariate (*r* = −0.33, *p* = 0.005).

### Additional exploratory analyses

Given that the strongest correlation between ToM and positive symptoms was with the item Lack of judgement and insight, and that there is a debate regarding whether that item could be better classified with the cognitive/disorganization dimension (43), we reexamined the correlations with ToM (both with and without the covariate) after excluding that item from the positive dimension. When including non-social reasoning as a covariate, the correlation between ToM and positive symptoms remained significant (*r* = −0.25, *p* = 0.039). Without the covariate, the correlation remained non-significant (*r* = −0.183, *p* = 0.129). When adding the item Lack of judgment and insight to the cognitive/disorganization dimension, the correlations were again significant with (*r* = −0.42, *p* < 0.001) and without the covariate (*r* = −0.48, *p* < 0.001).

## Discussion

The aim of this study was to examine the associations between ToM and the clinical dimensions of schizophrenia using a psychometrically sound measure of ToM (i.e., the COST) and the five dimensions of the PANSS including positive, negative, cognitive/disorganization, depression/anxiety and excitability/hostility symptoms in patients with recent onset SSD, while controlling for non-social cognitive abilities. An exploratory objective was to examine the associations between ToM and the individual symptoms included in the five dimensions of the PANSS. The present study was the first to investigate the relationship between clinical symptoms and ToM performance as assessed with the COST, which may allow the detection of more subtle ToM difficulties given its good psychometric properties and absence of ceiling effects (8, 31). Importantly, the COST also includes diverse control conditions, which allowed us to examine the link between ToM and symptoms while controlling for the non-social neurocognitive abilities involved in this type of tasks. The results showed that controlling for non-social reasoning had an important influence on the pattern of results, suggesting that some of the previously reported associations between ToM and symptoms may reflect general (non-social) cognitive abilities rather than social processes. More specifically, when controlling for non-social reasoning, a moderate negative correlation was observed between ToM and the positive symptoms dimension and a moderate to large negative correlation was found between ToM and the cognitive/disorganization symptoms dimension. When *not* controlling for the non-social abilities, the current study replicated the results found in previous studies for the significant correlations between ToM and negative symptoms (14, 20, 44, 45) and cognitive/disorganization symptoms (14), whereas the association with the positive symptoms dimension no longer reached significance. These results highlight the importance of the non-social cognitive abilities required to achieve good performance on story-based ToM tasks such as the COST and suggest that positive symptoms are more specifically linked to the social aspect of these tasks.

### Positive symptoms and ToM

The association between the positive symptoms dimension was statistically significant after controlling for non-social reasoning but did not survive the FDR correction when the association was examined without the covariate. This pattern suggests that positive symptoms could be more specifically associated with the social aspect of the task, and that this relationship may have been masked by more general deficits that were not accounted for in prior studies (20, 21, 23, 44). As previously mentioned, the link between ToM and positive symptoms is inconsistent in the literature, but some studies have nevertheless shown a significant association between these constructs (12, 13). These previous studies that reported significant associations with ToM generally targeted specific positive symptoms. For example, Mehl et al. (13) specifically targeted persecutory delusions and had an inclusion criterion of acute persecutory delusions by a minimum score of four for the PANSS item P6 (Suspiciousness and persecution). Moreover, they rated the severity of persecutory ideation with a measure specifically targeting this symptom, *The Peters and al. Delusions Inventory* (46). It is thus possible that more targeted assessments are more sensitive to detect an association between positive symptoms and ToM. It is also possible that targeting symptoms with a greater social component, such a persecutory delusions, could result in a greater link with ToM performance. In the current study however, the symptom Suspiciousness and persecution (PANSS P6) showed a very low, non-significant correlation with ToM performance. It is important to note that in our study, we targeted a sample of mildly ill patients (mean total PANSS score of 54.5) (36), a population for which it is particularly relevant to identify and understand the remaining barrier to functional recovery, including residual symptoms and ToM deficits. For the PANSS item P6, our participants had a mean score of 2.7, which is considerably below the minimum score of four used as an inclusion criterion in the study by 13. As a result, the association between ToM and specific positive symptoms such as persecutory delusions might only appear in a population with a greater severity of symptoms. On the other hand, it is possible that this relationship emerges in people with greater ToM deficits, which can influence their interpretations of social situations and thus contribute to persecutory delusions.

In this study, the examination of the individual positive symptoms revealed a significant correlation between ToM and the item Lack of judgment and insight, the significant effect only reaching the FDR corrected statistical threshold when including non-social reasoning as a covariate. A similar relationship between ToM and Lack of judgment and insight was also reported in several prior studies (17, 47, 48). For instance, Zhang et al. (48) showed that patients with poorer clinical insight performed worse on tasks of second-order ToM, whereas patients with adequate clinical insight performed similarly to healthy controls. Our study thus adds to this already recognized relationship between ToM and insight and suggest that this relationship could be even stronger once we account for the non-social processes involved in performing ToM tasks.

### Negative symptoms and ToM

The association between the negative symptoms dimension and ToM did not reach statistical significance when controlling for non-social reasoning, but was significant without the covariate. While it would be tempting to conclude that it is the cognitive demands of ToM tasks that drive the association with negative symptoms, the association between the negative symptoms dimension and our non-social reasoning control condition fell below statistical threshold (*p* = 0.054) in the current study. However, numerous prior studies have targeted the link between general (non-social) cognitive abilities and negative symptoms (19, 49–51), and this relationship is now well-recognized in the literature. Cognitive abilities have however not typically been considered when assessing the relationship between ToM and negative symptoms, and our study suggests an impact of non-social reasoning on the previously reported relationship between ToM and negative symptoms (14, 52, 53).

When looking at the individual symptoms within the negative dimension, the analyses without the covariate revealed significant associations with Emotional withdrawal, Poor rapport and Passive/apathetic social withdrawal. These symptoms are part of the experiential category of negative symptoms, as opposed to the expressive negative symptoms (54). Experiential negative symptoms refer to a diminished motivation and enjoyment for a range of activities, including social interactions (55). The current results indicate that these experiential symptoms are likely to lead to a reduced understanding of others' cognitive and affective states, perhaps through less occasions to learn about others' mental states.

### Cognitive/disorganization symptoms and ToM

The results of this study also showed that more severe cognitive/disorganization symptoms are significantly associated with poorer ToM performance, with or without controlling for non-social reasoning. Within this symptoms dimension, Abstract thinking and Conceptual disorganization were more specifically correlated with ToM, with or without controlling for non-social reasoning, whereas Poor attention only reached significance without the covariate. The significant association between ToM and the cognitive/disorganization symptoms dimension is in line with our hypothesis and with prior studies suggesting a relationship between ToM and neurocognition (11, 19), and more specifically with abstraction. In a meta-analysis exploring the relationships between ToM and neurocognition in schizophrenia, abstraction showed the strongest association to ToM among all executive functions (11). Abstraction is defined as the process of formulating general concepts by abstracting common properties of instances (56). The presence of cognitive/disorganization symptoms could make difficult the process of forming ToM meta-representations of one own's thoughts or the thoughts of others (14). Thibaudeau et al. (11) proposed that making a ToM judgment requires an integration of concrete information (e.g., facial expression, the context of a situation, etc.) to infer a mental state that is not explicitly presented. As a result, abstraction and ToM could share common processes, explaining the strong association existing between these two constructs (11), ToM thus being a social form of abstraction.

The association between Conceptual disorganization and ToM is consistent with the results of a meta-analysis by de Sousa et al. (57) which observed a moderate association between ToM and global ratings of thought disorder (*r* = 0.35). Conceptual disorganization is defined as disorganized process of thinking characterized by disruption of goal-directed sequencing (3) and is typically rated by a clinician or a research team member according to the patient's speech during the interview (e.g., SCI-PANSS) (40). Docherty et al. (58) suggested that ToM deficits could lead the patients to be unaware of what the listener needs in order to understand their speech (i.e., lack of awareness of the perspective of the listener). In addition, Frith (15) suggested that difficulties inferring the mental states of others or interpreting other's social signals during a conversation may prevent repair if a communication failure occurs, resulting in speech being perceived by the interlocutor as tangential or derailed. The association between Conceptual disorganization and ToM supports that SSD may conjointly affect the ability to understand others (i.e., ToM) as well as the ability to be understood by others (i.e., thought disorder or conceptual disorganization) (59–61).

### Anxiety/depression symptoms, excitability/hostility and ToM

In line with our hypothesis and previous studies (53, 62, 63), no significant association was found between ToM and anxiety/depression or excitability/hostility symptoms dimensions. Our sample was not highly symptomatic in these dimensions, with an average rating of 2.0 per symptom for anxiety/depression symptoms and 1.5 for excitability/hostility symptoms. Hence, a possible hypothesis is that the association between ToM and these symptom dimensions may only occur in a population with higher clinical severity.

### Clinical contributions

As documented in the literature, an important obstacle to adequate functioning in schizophrenia is the presence of social cognition deficits (26). Specifically, ToM has been shown to be one of the best predictors of daily functioning in schizophrenia, even in people with recent-onset of the disorder (5, 26, 29, 59, 60, 64). As such, a variety of interventions have been designed to directly address these deficits (65, 66). The current study sheds light on the associations between specific clinical symptoms of the disorder and performance in ToM. Our use of the five-dimensions version of the PANSS made it possible to target not only the positive and negative symptoms dimension of schizophrenia, but also the cognitive/disorganization dimension, which showed a moderate to strong correlation with ToM. Within this symptom dimension, Difficulty in abstract thinking, as the most strongly correlated individual symptom with ToM, could be of particular clinical interest as clinicians could learn to pay particular attention to these symptoms as a cue to orient the patients toward a more complete evaluation of their ToM abilities. The prompts provided to screen for Difficulties in abstract thinking in the Structured clinical interview for PANSS (SCI-PANSS) (40) could be a good resource for clinicians, this tool already being used is some first-episode psychosis clinics. Early screening and therapeutic management would be optimal for the patients, interventions being at their most effective in the first 5 years following the first psychotic episode to reduce the risk of long-term functional impairments (67, 68). These interventions include cognitive remediation, a behavioral training based intervention that aims to improve cognitive processes (attention, memory, executive function, social cognition or metacognition) with the goal of durability and generalization” (Cognitive Remediation Experts Workshop – CREW, 2010). There are several cognitive remediation interventions that currently exist to address both neurocognitive and social cognitive deficits in individuals with SSD (e.g., *Metacognitive Training, SocialVille Training Program, Social Cognition and Interaction Training*, etc.) (33, 69–71). For example, the *Metacognitive Training* and the *Social Cognition and Interaction Training* both include specific modules on theory of mind abilities (69, 71). Interestingly, cognitive remediation programs that do not explicitly target social abilities, for instance the program CIRCuiTS (Computerized Interactive Remediation of Cognition and Thinking Skills), can also improve ToM (72). A multiple case study showed significant improvements in ToM after providing CIRCuiTS, a cognitive remediation therapy that targets metacognition and basic cognitive skills (attention, memory, and executive functions) (72, 73). Furthermore, improvements were also observed in cognitive functions (e.g., executive functions, attention) and clinical symptoms (e.g., cognitive/disorganization symptoms, positive symptoms) measured by the five dimensions of the PANSS (5). These results further support that ToM and neurocognition are prime targets to improve clinical symptoms and functioning in individuals with SSD. For the disorganization symptoms (e.g., conceptual disorganization), it is unclear and less documented whether cognitive remediation programs also decrease the occurrence of these symptoms. Based on a systematic review and meta-analysis, de Sousa et al. (57) identified a knowledge gap such that we do not know much about the impact of cognitive remediation programs on the improvements of thought disorder (conceptual disorganization symptoms). New cognitive remediation modules could hence be developed to focus more directly on perspective taking in communication.

### Limitations

This study has three main limitations. First, the sample was overrepresented by men (87.1%), limiting the generalizability of the current results to women. In prior studies, men seemed to present with worse negative and less depressive symptoms than women (74, 75). Therefore, the severity of the negative symptoms could have been reduced if there was a greater proportion of women in the sample. However, the meta-analysis by Thibaudeau et al. (19) did not find a significant influence of the sex of the participants on the association between ToM and symptoms. This question would however deserve more direct assessment in a sample including a greater proportion of women. Second, the sample was composed of stable outpatients with recent onset of SSD who showed relatively mild levels of symptoms. While we nonetheless observed some significant associations between symptoms and ToM, it would be interesting to see if we can replicate the results of the current study in inpatients or patients with a more chronic course of SSD. Third, the use of a correlational study design does not allow causal links to be drawn. Therefore, the current study cannot conclude whether ToM deficits lead to increased symptom severity or vice versa. Finally, due to the small magnitude of the change in correlation strength between the analyses including or not non-social reasoning as a covariate, the shifts from significant to non-significant should be interpreted with caution.

## Conclusion

A thorough understanding of the relationships between positive symptoms, negative symptoms, cognitive/disorganization symptoms and ToM is essential to the development of personalized treatments that focus on recovery from SSD. The current study was the first to explore the associations between ToM and clinical symptoms using the COST, a task presenting with good psychometric properties and comprising control measures. Moreover, using a five dimensions approach is a more optimal way to categorize the different symptoms of SSD, likely leading to more valid results.

## Data availability statement

The original contributions presented in the study are included in the article/supplementary material, further inquiries can be directed to the corresponding author.

## Ethics statement

The studies involving human participants were reviewed and approved by Comité d'éthique de la recherche sectoriel en neurosciences et santé mentale du CIUSSS-CN. The patients/participants provided their written informed consent to participate in this study.

## Author contributions

AC wrote the protocole, conducted the analyses, and wrote most of the article. ÉT, CC, and AA contributed to the analysis and the writing of the article as well as the reflective process behind it. Throughout this study, M-AR collaborated with the authors and contributed to the article's revision. All authors contributed to the article and approved the submitted version.
